# The Head-to-Toe Hormone: Leptin as an Extensive Modulator of Physiologic Systems

**DOI:** 10.3390/ijms23105439

**Published:** 2022-05-13

**Authors:** Monica Misch, Prasanth Puthanveetil

**Affiliations:** 1 Chicago College of Osteopathic Medicine, Midwestern University, Downers Grove, IL 60515, USA; monica.misch@midwestern.edu; 2 Department of Pharmacology, College of Graduate Studies, Midwestern University, Downers Grove, IL 60515, USA

**Keywords:** leptin, metabolism, systemic role, physiological functions, leptin receptor

## Abstract

Leptin is a well-known hunger-sensing peptide hormone. The role of leptin in weight gain and metabolic homeostasis has been explored for the past two decades. In this review, we have tried to shed light upon the impact of leptin signaling on health and diseases. At low or moderate levels, this peptide hormone supports physiological roles, but at chronically higher doses exhibits detrimental effects on various systems. The untoward effects we observe with chronically higher levels of leptin are due to their receptor-mediated effect or due to leptin resistance and are not well studied. This review will help us in understanding the non-anorexic roles of leptin, including their contribution to the metabolism of various systems and inflammation. We will be able to get an alternative perspective regarding the physiological and pathological roles of this mysterious peptide hormone.

## 1. Introduction

The discovery of leptin arose simply from suspicion. Researchers noted than an isolated mutant mice colony lacking the *ob* gene possessed abnormal characteristics, such as hyperphagia, decreased energy expenditure, and early-onset obesity [1]. In 1953, a theory proposed the existence of a circulating molecule secreted by adipose tissue; this molecule was in direct communication with the hypothalamus and affects food intake, body weight, and long-term energy balance [2]. It was not until forty years later that this speculated molecule was characterized and identified. Derived from the mRNA transcript of the *ob* gene, this peptide hormone was comprised of 167 amino acids and named “leptin”, from the Greek work “lepto”, meaning “thin” [3]. Since its discovery, leptin’s pleiotropic effects have been found to influence hematopoiesis, angiogenesis, blood pressure, bone mass, and T-lymphocyte function, among other things [1,3]. The perception of leptin just as a peptide hormone that regulates body weight has evolved to now being seen as a signaling molecule capable of regulating physiological homeostasis [1,3].

## 2. Origins and Expression

In humans, the *ob* gene is expressed primarily in adipocytes; thus, serum leptin concentration is highly correlated to overall fat content in infants, children, and adults [4,5]. Leptin expression was found to be nearly double in subcutaneous fat relative to visceral and omental fat, and this principle has been proven true for both lean and obese individuals [4,5]. Leptin circulates either freely or bound to the extracellular portion of its receptor [5,6,7,8,9,10,11,12]. Binding of leptin on to its receptor allows it to cross the blood–brain barrier and admit access to the central nervous system. Free leptin has also demonstrated to have high blood–brain permeability, a process facilitated by tanycytes under conditions of capillary leaks and during debilitated blood–brain barrier integrity [5,6,7,8,9,10,11,12].

Although six isoforms of the leptin receptor have been identified, the primary receptor is the long form and resides in the arcuate nucleus of the hypothalamus [10,13,14,15,16]. If the neurons housing this receptor bind leptin, the receptor dimerizes and initiates a signaling pathway via cytoplasmic tyrosine kinase such as janus kinase (JAK) [10,13,14,15,16]. Following phosphorylation of the intracellular region of the receptor, the STAT proteins (STAT2 or STAT3) house Src-domains that allow them to anchor to the receptor [9,10,12,13,14,15,16,17,18,19]. Once activated, the STAT protein travels to the nucleus to initiate transcription of the pro-opiomelanocortin (POMC) gene; POMC has been identified as an anorexigenic peptide [20,21,22]. In addition, leptin inhibits neurons expressing the antagonist for POMC and neuropeptide Y (NPY) [20,21,22,23,24,25]. A lepti- mediated decrease in NPY also contributes to an anorexic effect, as NPY is a potent centrally acting orexigenic agent [20,21,22,23,24,25]. Fluctuations in leptin levels during fasting or starvation is a crucial factor [20,22,23,24,25]. In a normal, “fed” state, leptin levels are proportional to the amount or mass of adipose tissue in the body [20,22,23,24,25]. Aside from an individual’s baseline leptin levels, serum leptin can increase as much as 40% following an episode of overeating or decrease by 60 to 70% following chronic fasting [26,27,28]. Clearly, leptin is in direct communication with the central nervous system to flag acute changes in energy intake [26,27,28].

In addition to the CNS regions, there is also distribution of leptin receptors in the peripheral tissues [29]. This review also sheds light upon the role of leptin receptors across the physiological system, and the role of their accompanied signaling in regulating physiological functions.

## 3. Gastrointestinal System

The outreach of leptin expression extends into the gastrointestinal system and is continuous in the stomach. Leptin expression was greatest in the fundic region, where chief cells and parietal cells exhibit high reactivity to the hormone [30,31,32]. It should be noted that leptin receptors were detected in both healthy gastric epithelium and cancerous gastric cells [30,31,32]. With respect to physiological control of leptin secretion, high fat diets play an important role. Arita et al. [33] identified that a greater quantity of gastric leptin receptors became phosphorylated with consumption of a high fat diet [33]. The findings confirm that leptin secretion and leptin signaling are elevated on such a diet. Tracking down the GI tract, this report finds the link between leptin and intestinal health [33]. Mice lacking the leptin receptor were protected from intestinal dysbiosis and high-fat-diet-induced intestinal metaplasia, which reinforces the link between leptin secretion and gut health [33]. In the colon, a greater concentration of leptin receptors were found in ulcerative colitis patients despite serum leptin levels being normal [34,35]. It is worth considering in future research endeavors the density of leptin receptors in the GI tract, rather than just focusing on serum leptin levels. These findings characterize serum leptin as a mark of *localized* inflammation, especially in the gut.

## 4. Pancreas

There is an intimate and rather complex relationship between leptin and the secretory capacity of the pancreas. Leptin signaling induces the K+/ATP membrane receptor. The ultimate effect achieved is the conductance of K+ increases across the membrane, which hyperpolarizes islet cells to inhibit insulin secretion [36,37,38,39]. Leptin prevents the secretion of both insulin and glucagon [36,37,38,39]. While receptors for leptin are copious on β-islet cells and δ-islet cells, they are absent from the majority of glucagon-producing α-islet cells [36,37,38]. Thus, the inhibitory influence of leptin is more profound for insulin when compared to glucagon [39,40,41]. Please note that no profound effects on somatostatin secretion are observed following administration of leptin. Insulin signaling and leptin have got an interesting interaction that suggests a bi-directional feedback loop; as the secretion of insulin is known to stimulate leptin release, the released leptin limits insulin levels [39,40,41]. In this light, leptin can be considered as a regulating hormone for the pancreas. Another hypothesis for leptin’s inhibitory effect on insulin secretion is via the activation of intracellular cAMP signaling [42]. Leptin was shown to inhibit cAMP activity, and as a result, prevent the insulin secretory mechanism in the pancreatic cells [39,40,41]. An attributed mechanism is due to activation of phosphoinositide 3-kinase-cyclic nucleotide phosphodiesterase 3B (PDE3B) signaling nexus [43,44]. Activated PDE3 B could lead to enhanced breakdown of cAMP, mitigating the signaling responsible for pancreatic insulin secretion [43,44]. An additional possibility to consider is the toxicity of inflammatory cytokines and adipokines, which are induced or triggered by leptin and could act on β-cells to further compromise the ability to effectively secrete insulin [43,44]. Perhaps future investigation of the direct and indirect effects of leptin signaling on pancreatic islets would provide clarity.

## 5. Hepatic Tissue

Within the liver, leptin is considered an anti-steatosis hormone, but the levels and duration matters. The signaling cascade that leptin elicits in the liver targets a specific transcription factor that is a key component in lipid synthesis; its modulation by leptin mobilizes lipids [45]. The protective properties against lipid accumulation within the liver can be illustrated by observations made in models devoid of leptin receptors and accompanied by increased liver triglycerides and increased lipid deposition [46]. Moreover, leptin-deficient animal models saw a delay in liver regeneration, hypothesized to be a consequence of impaired angiogenesis and glucose transport to hepatocytes [47]. Although increases in circulating leptin would be presumably helpful, there seems to be a threshold that exists where chronic elevations exacerbate inflammatory and fibrogenic processes in the liver [45]. In fact, leptin was required for fibrosis to develop in mouse models with chronic liver injury [48]. Provided that the leptin receptor is expressed by Kupffer cells, hepatic stellate cells, and sinusoidal endothelial cells, there seems to be a direct mechanism of action for the development of fibrosis [45]. Likewise, the leptin-dependent induction of fatty acid oxidation and mitochondrial respiration taking place in the liver may induce oxidative stress [45]. When this fragile balance between leptin signaling and lipid mobilization becomes dysfunctional, individuals may be susceptible to pathological conditions. A positive correlation has been established between elevations in circulating leptin in non-alcoholic fatty liver disease patients, steatosis patients, as well as non-alcoholic steatohepatitis patients (NASH) [49]. It is important to consider the levels of leptin were influenced by factors such as age, gender, co-existing metabolic diseases, and percentage of body fat. Although increased leptin is not a direct indicator of these pathologies, it raises curiosity about its role as an indicator of ongoing metabolic complications.

## 6. Connective Tissue

In skeletal system, leptin is known to be a potent inhibitor of bone formation [50,51]. Bone loss becomes a concern for those with substantial fat stores, or in other words, subjects who present with an elevated body mass index, since there is a greater availability of fat cells to synthesize the hormone leptin. Leptin is also a potent inhibitor of bone formation [50,51]. Bone loss becomes a concern for those with substantial fat stores, elevated body mass index, and especially subjects who are insulin-resistant or have type 2 diabetes. A study evaluated the long duration effects of leptin by administering recombinant adeno-associated virus-rat leptin (rAAV-Lep) into the third ventricle of the hypothalamic region to understand the impact on weight gain and bone metabolism using female Sprague-Dawley rats which had sufficient leptin levels [52]. Interestingly in this study, at 5 weeks after vector administration, rAAV-Lep-administered rats developed lower cancellous bone volume and bone marrow adiposity. With the increase in duration of treatment, no significance differences were noted in cancellous bone but a major impact on bone adiposity and associated weight gain [52] was seen. Another study demonstrated that intracerebroventricular (ICV) administration of leptin reduces trabecular bone volume, most notably in the vertebral column [53,54,55]. It is unclear whether this loss of bone volume is due to the direct action of leptin on bone or its influence on the sympathetic nervous system (SNS). We also have found reports that demonstrate contradictory effects. For instance, leptin binding to its receptors on osteoclasts and osteoblasts elicits synthesis of the bone matrix. Leptin promoted the differentiation of osteoblasts, synthesis of Type I collagen, and allocation of osteocalcin [56]. Leptin by itself and in the presence of cytokines has been demonstrated to enhance collagenolytic and gelatinolytic properties in bovine cartilage explant cultures [57]. Leptin brings about this effect through the simultaneous activation of multiple matrix metalloproteinases (MMP1 and MMP13) and also involving transcription factors such as signal transducers and activation of transcription of family members (STATs) [57]. These effects were nullified by using an anti-leptin antibody [57]. Leptin inhibited osteoclast formation by increasing concentrations of osteoprotegerin, a protein that inhibits maturation of osteoclasts [58]. Aside from acting peripherally on bone, leptin’s activation of the ventromedial hypothalamus may have an indirect consequence of activating noradrenergic signaling at the osteoblasts, mediating its ability to impact bone mass [53,54,55]. It is difficult to isolate the effects of leptin on the skeletal system when leptin also acts alongside hypothalamic effectors, such as cortisol, IGF-1, estrogen, and parathyroid hormone [59]. Thus, the link between ICV administration of leptin and reduced trabecular bone sparks inquisition. Given that leptin has pro-osteogenic properties alone, there must be a complicated pathway that creates the overall consequence of reduced bone volume in hyperleptinemic cases that needs further evaluation.

## 7. Circulatory System

Once activated, T cells express a leptin receptor on their membrane, and when the receptor is revealed, the T cells become sensitive to changes in insulin concentration and nutrient availability [52,53]. Given that T-cell activation is energetically expensive, levels of serum leptin reflect nutrient availability for the process to begin [52,53]. The signaling pathways found to be mediated by the leptin receptor in T cells include upregulation of glucose intake, optimization of lactate production, proliferation, and production of inflammatory cytokines [52,53,54,55,56,57]. Additionally, the ability of T-cells to secrete inflammatory cytokines IL-2 and IFN-γ was found to be dependent on leptin availability [54,55,56,57]. Although leptin may be necessary for mounting a typical immune response, elevated serum leptin is a high-risk factor for many hematopoietic malignancies [58,59,60,61,62]. This was found to be true when leptin receptors were absent in normal promyelocytes, but leukemic promyelocytes housed mRNA of multiple isoforms of the leptin receptor [58,59,60,61,62]. The cascade of STAT3 and ERK 1/2 signaling that follows leptin receptor activation resulted in increased colony-forming ability, proliferation, and anti-apoptotic properties of human erythroleukemic cell lines. These findings are significant because they illustrate the direct effect of leptin on the pathological progression of hematopoietic malignancies [60,61,63]. A crucial note to make is that for certain blood cancers, serum leptin was elevated independent of the patient’s BMI [60,64], persuading against confounding factors such as obesity. These findings suggest focusing on the dysregulation of leptin itself, or leptin-mediated pathways as treatment for certain leukemias rather than focusing on BMI. To further highlight leptin’s role in the development of leukemia, leptin receptor mRNA was constitutively expressed in acute myelogenous leukemia and acute lymphocytic leukemia; leptin receptor expression also correlated well with immature CD34+ hematopoietic progenitor cells [59,60,65]. The above-mentioned findings express the importance of leptin during the blast or proliferative stage of blood cancers, rather than in more chronic stages. Early intervention and modulation of leptin signaling has the potential to be a promising route for leukemia research.

More recent studies in healthy patients point to leptin regulating blood flow and have identified a saturable, designated binding site for leptin on red blood cells [66]. The researchers found that leptin ultimately induced an increase in red blood cell-derived ATP, a recognized stimulus of blood flow [66]. Furthermore, leptin is also known to cause nitric oxide release and consequent vasodilation in endothelial cells [66]. Although leptin is known to play a crucial role in developing hematopoietic malignancies, it may also be beneficial to the circulatory system under normal physiological conditions.

## 8. Cardiovascular and Renal System

Leptin’s effects on the cardiovascular system are discordant and not well understood. Although both large population-based and clinical studies have found a positive correlation between hyperleptinemia and cardiovascular complications [67,68,69,70,71], it is unclear whether the adverse events are driven by hyperleptinemia alone. The accumulation of white adipose tissue that contributes to hyperleptinemia has other physiological consequences, such as obesity, hypertension, and diabetes and that could act as confounding factors for the cardiovascular events, as per the studies done in human subjects [67,68,69,70,71]. Regardless, it is clear that leptin has the potential to play a role in cardiovascular health. Leptin receptors have been located on hemopoietic cells [61,72,73,74,75,76], rightfully characterizing leptin as a systemic signaling molecule. Previous experiments have identified leptin as a promoter of platelet aggregation as well as an accelerator for wound repair, as per the observations done in human subjects [77,78,79,80,81,82,83,84,85,86]. These findings are evidence for leptin’s ability to facilitate the onset of thrombotic events or stroke in human subjects, which contributes to the growing interest of its role in regulation of the circulatory system. Likewise, leptin receptors have been identified in human atherosclerosis [87,88,89], which highlights the role of leptin signaling in endothelial dysfunction [87,88,89]. Leptin signaling has also shown to contribute towards hypertension [90,91,92,93,94,95] in mice and rats, an effect mediated by angiotensin II [90,91,92,93,94,95].

The endothelial cells have been demonstrated to have substantial leptin receptor gene expression. With activation of the leptin receptor, a tyrosine-kinase-dependent pathway initiates angiogenic processes [96,97,98,99] in human and animal cell model systems. Interestingly, Kang et al. [100] found that atherosclerotic lesions in human subjects had a greater expression of the leptin receptor gene when compared to histologically normal endothelium [100]. Note that obesity is identified as a major risk factor for atherosclerosis [101]. It would be logical to consider that the excess adipose stores in an obese individual could contribute to hyperleptinemia. Increased leptin levels have shown positive correlation with increased blood viscosity and enhanced platelet count with fibrinogen expression and activity [102]. This could explain leptin’s role in aggravating atherosclerotic lesions. 

Furthermore, leptin has been demonstrated to enhance the sympathomimetic effect, thus raising peripheral blood pressure [92]. This is not a mere correlation but supported by evidence as lean individuals who received exogenous leptin exhibited hypertension [103]. This illuminates the hormone’s ability to contribute to cardiovascular health independent of other contributing factors. In contrast, leptin’s signaling pathways do not always result in adverse outcomes; elevated serum leptin was also linked to cardioprotection [104]. Evidence from clinical trial has shown that leptin concentration was inversely associated with left ventricular and left atrial masses [101]. Further investigation is encouraged to determine if these effects are occurring through a separate signaling pathway or a mediated by its own receptor isoforms. Although some discrepancy still exists, there is a consensus that both excessive leptin and leptin deficiency would have an impact on cardiovascular health.

As a large molecular weight protein, leptin can be problematic for renal filtration. Hyperleptinemia is associated with impaired kidney function, including increased excretion of urinary albumin and a reduced glomerular filtration rate [96,97,98,99,100] in patients with chronic kidney diseases (CKD). Leptin is considered a uremic toxin, as elevated levels are associated with glomerular mesangial cell hypertrophy, fusion of podocytes, reduced metabolic activity in the proximal convoluted tubule, and thickened basement membrane [96,97,98,99,100], as observed in CKD patients. These consequences contribute to albuminuria, glomerular sclerosis, and apoptosis of nephrons.

## 9. Nervous System

On a biochemical level, leptin triggers anorexigenic neurons in the hypothalamus to synthesize pro-opiomelanocortin (POMC) and cocaine and amphetamine-related transcript (CART), which are two polypeptides that are known to limit food intake and increase energy expenditure [105,106,107]. Leptin has the power to simultaneously inhibit orexigenic neurons from synthesizing agouti-related-peptide (AGRP) and neuropeptide Y (NPY), which have antagonistic effects on satiety and promote feeding behavior [102,108,109] in both rats and mice. In other areas of the brain, leptin influences the lateral hypothalamus to decrease the expression of orexins, or general neuropeptides involved in food regulation and stress. Leptin is also known to directly activate a transcription factor called Steroidogenic factor-1 (SF-1) on neurons of the ventromedial hypothalamus regions [110,111] in rodents. When mice with leptin receptors knock down in generated SF1 positive neurons, these mice gained weight due to the loss of restriction on weight gain by leptin receptors [110,111]. It is evident that leptin is at work in many areas of the brain to control orexigenic urges as well as energy use. Additionally, the ventral tegmental area of the brain contains neurons possessing the leptin receptor. Leptin signaling in this region is a well-validated pathway involved in suppression of hunger [110,111]. Leptin receptors are ubiquitously expressed across astrocytes and microglia as well, which are targets for pro-inflammatory signaling within the hypothalamus [105,106,112,113,114,115]. The exact role of astrocyte and microglial residing leptin receptor needs to be understood further.

## 10. Immune System

Leptin serves as a communication link between the metabolic and immune systems [107,116,117]. The formation of a sufficient line of defense against pathogens is a highly energy-dependent process [118,119,120,121]. Thus, the presence of leptin receptors on most immune cells represents a close interplay between the body’s metabolic status and its ability to mount an immune response. With respect to innate immunity, leptin increases the cytotoxicity of natural killer cells, as well as increases the activation of granulocytes, macrophages, and dendritic cells [122,123,124,125,126,127,128]. As for adaptive immunity, leptin limits the proliferation of regulatory T cells but increases the production of naïve T cells and B cells [53,129,130,131,132,133]. Although the exact mechanism is unclear, the presence of leptin is believed to influence cell survival, as exogenous leptin was found to delay apoptosis via intracellular JAK, NF-kB, and MAPK pathways [134,135,136]. Overall, leptin induces an inflammatory response via immune cell activation, inducing chemotaxis and the release of cytokines [53,129,130,131,132,133]. Likewise, leptin plays a role in immunity by maintaining the balance of Type I and Type II Helper T cells [137,138,139]. Without sufficient levels of leptin, the ability to create CD-4 cells is compromised [137,138,139].

## 11. Sexual Dimorphism and Leptin

### 11.1. Sex-Specific Effects of Leptin: On Females

Serum leptin levels rise and fall throughout a woman’s menstrual cycle. In fact, estrogens induce leptin release [140,141,142,143,144,145,146]. The rise of estrogen that peaks mid-cycle is accompanied by a mid-cycle peak in leptin [140,141,142,143,144,145,146]. No studies have been done to confirm an ovarian contribution to serum leptin, but it seems that leptin levels can be used as a direct measurement of ovarian follicular health and its ability to produce other hormones, such as progesterone and LH. Based on observation in human subjects, during the menses phase, the level of leptin is close to or slightly over 15 ng/mL. During the follicular phase, the concentration rises to be over 15 ng/mL but less than 20 ng/mL [147]. During the ovulatory phase, leptin levels peak along with estrogen levels and reach approximately mid 20s (>20 ng/mL) [147]. With the luteal phase, the peak starts to decline and reaches back to the level of 20 ng/mL [147].

Leptin’s interaction with progesterone and LH remains ambiguous. Although leptin and progesterone show similar patterns of serum fluctuations during a healthy menstrual cycle [140,141,142,143,144,145,146], there is no evidence of regulation at a pre or post-translational level. With respect to LH, leptin receptor activation induces the STAT3 signaling pathway. It is the STAT3 induction, rather than leptin itself, that is responsible for the LH surge [148,149,150]. Nonetheless, leptin contributes directly or indirectly to the regulation of the reproductive cycle. 

The female reproductive system is a hallmark example of the need for research surrounding leptin expression. Normally, mammary epithelial cells have moderate expression of the leptin receptor gene. However, carcinoma cells within mammary epithelium showed a significant increase in leptin receptor expression [147,151,152]. It is important to note that these cells produce leptin themselves, more so than the non-cancerous control. This points to an autocrine signaling mechanism that may contribute to the proliferation and metastasis in breast cancer populations [147,151,152]. Interestingly enough, the tumors did not metastasize if they lacked the leptin or the leptin receptor gene [147,151,152], which confirmed the hypothesis. More recent studies confirm the link between overexpression of leptin and its receptor in both primary and metastatic cancers [153,154,155,156,157]. These findings stress the importance of a healthy BMI and fat content in cancer prognosis. Likewise, it also illustrates how obesity can be a detrimental factor for patients diagnosed with cancer, due to metabolic effects and also leptin-mediated direct effects.

### 11.2. Sex-Specific Effects of Leptin: On Males

Even though leptin receptors have been identified in the testes [158,159,160,161,162,163], the effects of leptin on the male reproductive system are less explored. Recent studies in rat models have shown that leptin is a direct inhibitory signal for testicular steroidogenesis [164]. Associations between high BMI, hyperleptinemia, low serum testosterone, and impaired sperm motility have been identified but not confirmed [158,159,160,161,162,163]. Serum leptin concentration following fasting has been shown to be lower in males (approximately >6.5 ng/mL compared to over 15.0 ng/mL) in comparison to females, suggesting females have a higher potential to generate leptin from comparable fat mass [147,165]. Interestingly, there has been reports that for females, there is a permanent drop of total leptin below 20 ng/mL in the post-menopausal stage [147,165]. Even for males, during their active adult life (30–50 years), the levels of leptin have been reported to be just over 10 ng/mL, permanently dropping to a level of just above 6 ng/mL after 50 years of age [147,165].

As such, the inhibitory influence of adipocyte leptin on androgens raises concern for elevated BMI values and infertility. Additionally, the role of leptin receptor stimulation by leptin released from both testicular and extra testicular tissues has not been well studied and needs attention. Definitely, enhanced plasma leptin levels have been well associated with both prostate cancer and testicular cancer in males, and the leptin receptor is a known target for treating these cancers in the male population [166,167,168]. There is also evidence that leptin is not a robust biomarker in males in comparison to females with the same types of cancer [169]. In lung and hematological cancers, the leptin levels in females are shown to have over 30 ng/mL, and for gastrointestinal and genitourinary cancers, the levels are over 20 ng/mL, in comparison to males, where the levels are less than 10 ng/mL [169]. In comparison to healthy conditions, the plasma levels are still high in males following cancer [169]. These observations can help us in drawing conclusions that leptin could be considered as a marker for cancer in males and an even more robust marker in females.

## 12. Leptin and Systemic Health

### 12.1. Overall Systemic Metabolic Homeostasis

This section will highlight on the divergent effects of leptin, which may not fit into a single organ system. The identification of leptin as a key player in metabolic homeostasis is rooted in its systemic effects when one abstains from eating. When leptin levels fall as a result of a fasted state, there is a neuroendocrine shift that promotes increased appetite with a concomitant effect of decreased energy use [140,141,142,143,144,145,146,149]. This overall effect is achieved by reducing testosterone, TSH, and the loss of LH hormone cycle in females [146,165,166,167,168,169,170,171,172,173,174,175,176].

Furthermore, leptin can induce the expression of insulin-like growth factor binding protein [177,178,179,180]. The mechanism is described by Won et al. as being direct and indirect. Leptin can directly and indirectly stimulate the expression of IGF-1 and IGF-2, based on the evidence from reported studies [177] in a teleost fish model. As a result, there is an enhanced glucose uptake and glycogen synthesis across the periphery. In skeletal muscle, leptin signaling could initially cause an increase in lactate production, but in contrast, it is important to understand that chronic leptin could also decrease muscle triacylglycerol accumulation [181,182,183,184,185,186], as per the observations from porcine myoblasts and rat and mice skeletal muscle tissues. 

#### Leptin Has a Crucial Role in Carbohydrate Metabolism

With respect to cellular glucose uptake, leptin shares many of its intermediate signaling pathway with insulin. The overlap begins at the level of phosphatidylinositol-3 kinase, and both hormones initiate the process of GLUT4 expression in skeletal muscle [187]. Since the two hormones work together to produce similar effects, the isolated actions of leptin are an ongoing investigation and is really tough to dissect. In the presence of normal levels of insulin and glucagon, leptin treatment was found to increase the expression of GLUT4 transporters up to two-fold [188]. These findings suggest leptin is an enhancer of glucose uptake and insulin sensitivity in skeletal muscle and also a negative regulator for GLUT4 recruitment, TBC1D1 and TBC1D4 [189], facilitating these effects. It is important to note that when insulin was removed as a confounding variable, leptin was not able to upregulate the insulin-stimulated uptake of 2-deoxyglucose or glycogen synthesis [190]. It appears that insulin has a permissive effect on leptin, and leptin cannot achieve the physiological effects mediated by insulin in the absence of an active insulin signaling. The combination of insulin and leptin can increase glucose oxidation up to six-fold compared to a control or an unstimulated state, whereas either hormone on its own displayed comparable increases in glucose decarboxylation reactions [190]. Leptin’s target(s) in the carbohydrate metabolism pathways are unclear, but previous research reports have offered some insight. Leptin was found to increase pyruvate dehydrogenase activity and activity of the Krebs cycle. These findings were significant and even higher than that observed with insulin *per se* [190]. The aforementioned evidence validates a crucial role of leptin in systemic carbohydrate metabolism, but the extent to which it is insulin-independent is unknown and needs more clarity.

In the liver, leptin has been shown to have a negative effect on gluconeogenesis [191,192]. Also, synthesis of cholesterol and bile acids are also known to be modulated by leptin either directly or through the central effects [187,190,193,194]. Acute and chronic leptin has been shown to have differential effects on fatty acid uptake and utilization [188,189,195,196,197]. Due to the ability in regulating glucose, fatty acids, cholesterol, and bile acids, leptin is considered as a crucial regulator of metabolic and systemic health.

### 12.2. Leptin Imbalance and Associated Diseases

A reduction in adipose tissue mass is inevitable when daily energy expenditure exceeds energy intake. When one’s adipose tissue mass falls below a certain threshold and leptin levels are consequently decreased, dysregulation of the HPA axis will ensue [198]. The cessation of menstrual periods, along with an elevated risk of osteoporosis, is linked to hypoleptinemia [199,200,201,202,203]. Although it is not confirmed that leptin is the sole contributor to these manifestations of decreased female hormone levels, it is considered a necessary factor [199,200,201,202,203]. Leptin replacement therapy was successful in restoring healthy menstruation cycles in those with adipose mass below an optimal threshold, signifying the importance of this hormone [202,204,205].

Congenital leptin deficiencies exist, even though it is rare [206]. Besides obesity and presence of excess adiposity, leptin deficiency also results in decreased insulin sensitivity, unfavorable lipid profile, and hepatic steatosis [9,173]. Exogenous leptin administration has been confirmed to improve all metabolic parameters and is the first line of treatment in these individuals [173].

In contrast, hyperlipidemia conditions are a growing concern for chronic myocardial health and homeostasis [101,104]. Elevated circulating leptin potentiates atherogenic factors, including inflammation, hypertrophy, platelet aggregation, proliferation of vascular smooth muscle, formation of reactive oxygen species, and endothelial cell dysfunction [77,78,79,80,81,84,191,194]. Hyperlipidemia goes hand in hand with obesity-related diseases, making it a major risk factor for atherosclerosis and heart disease [84,191,194]. Polyakova et al. confirmed prolonged hyperleptinemia led to an increase in blood pressure, heart rate, myocardial hypertrophy, systemic inflammation, and frequency of ischemic arrhythmias [207].

Given the anorexigenic effects of leptin in the brain, it has become a strong contender in the treatment of obesity. Despite efforts to reduce weight gain with exogenous leptin, there is failure to generate a physiological response in obese patients [208]. The term “leptin resistance” is used to explain the absence of expected physiological response to hyperleptinemia in these obese patients [208]. It suggests that hyperleptinemia may be a driving force for obesity, as chronic treatment with exogenous leptin that exceeds the individual’s required limit significantly increases body weight [208]. The nature of hyperlipidemia is also dependent on diet composition, which highlights the multifactorial aspects of metabolic management [209]. In mouse models, only a high-fat, high-sugar diet increased serum leptin values without a corresponding increase in NPY mRNA expression; thus, even though leptin was elevated, the mice remained hyperphagic [209]. This is a salient finding because it suggests both high-sugar and high-fat diets could be possible factors for leptin resistance. It opens the door for new research to find other possible factors that may introduce leptin resistance.

### 12.3. Genetic Predominance Affecting Leptin Resistance and Its Role in Obesity

Genetics play an important role in inducing obesity and leptin resistance [210,211,212]. Some of the common genes that contribute towards obesity and subsequently leptin resistance are: mutations in leptin (LEP), leptin receptor (LEPR), Melanocortin 4 receptor (MC4R), Proopiomelanocortin (POMC), Brain-derived neurotrophic factor (BDNF), Proprotein convertase subtilisin/kexin type 1 (PCSK1), and peroxisome proliferator-activated receptor (PPARs) [213,214,215,216,217,218,219]. Broadly, all these mutations have been associated with hyperphagia, metabolic dysregulation, and altered gut brain signaling, followed by weight gain and insulin resistance [210,211,212]. Excess circulatory leptin levels, along with defective leptin receptor signaling, could lead to leptin resistance, which further aggravates obesity, allowing for the initiation of a vicious positive feedback loop [213,214,215,216,217,218,219]. Roughly around eight different mutations have been reported in the leptin gene and with leptin receptors, and few single nucleotide polymorphisms have been reported either in cytokine homology domain or in their fibronectin type 3 domain [210,211,212]. MC4R acts as a major mediator in CNS for the anorexic effect of leptin [213,214,215,216,217,218,219,220,221,222]. To date, over 370 single nucleotide variations have been reported for MC4R, and among these over 65 variations have been predicted to be highly pathogenic in clinical subjects [213,214,215,216,217,218,219]. Even though not frequent, the monogenic mutations form the predominant genetic reason for causing obesity and leptin resistance in early childhood [220,221,222], which contributes to childhood obesity. Most of the above-mentioned gene mutations are known to influence leptin and its associated receptor signaling leading to pathogenesis of childhood obesity with severe metabolic complications [210,211,212]. A detailed understanding of mutations in these targets could help in alleviating childhood obesity.

## 13. Leptin as a Diagnostic and Therapeutic Tool

### 13.1. Diagnostic Tool

Fluctuations in the expression of leptin and its receptor in various disease conditions raises the possibility of its potential as a diagnostic biomarker. One example is using serum leptin as an additional anthropometric index to classify obesity. A study has revealed that elevated levels of serum leptin were positively correlated to standard markers of obesity and showed the strongest correlation with hip circumference [210]. Currently, body mass index remains the standard for classifying individual obesity, but this value has been identified as an imperfect representation of fat mass [211]. Routine measurements with serum leptin concentration may provide a more accurate depiction of individual fat mass, as long as further studies establish appropriate cut-off points for normal, overweight, and obese patients. Another example of the use of leptin as a biomarker can be found in dermatology. Significant deviations from normal serum leptin concentration are currently being investigated in psoriasis. Elevations in serum leptin are being used as a biomarker for both the diagnosis and severity of psoriasis [212,213]. One must consider serum leptin is not sufficient to make a diagnosis alone, but at the same time, its use as a diagnostic marker may aid physicians in solidifying a differential diagnosis.

Further, research is underway to investigate the use of leptin as a biomarker of malignancy. Serum leptin has been found to be significantly elevated in cases of prostate cancer and breast cancer, independent of obesity [214,215]. Surpassing the mere detection of cancer, leptin expression was significantly correlated to the stage of metastasis, as well as the degree of lymph node development [216]. With respect to colorectal cancer, immunohistochemical measures of leptin were used to accurately predict the cancer prognosis, independent of other indicators [216]. These findings were significant as it introduces leptin as a marker of clinical outcome. It is important to note that analysis of leptin can also be done using patient saliva. In the first study of its kind, researchers identified leptin as a preoperative indicator of parotid tumors; salivary leptin was used to distinguish tumor patients from healthy individuals [217]. The value of leptin as part of a cancer diagnostic workup is an interesting avenue to pursue.

Leptin may be used in conjunction with other hormones or cytokines to elevate its diagnostic value. For example, the ratio of leptin to adiponectin (or the inverse) is of interest [218]. These two hormones have contrasting effects on the manifestation of inflammatory processes, and thus the development of metabolic syndrome. Although metabolic syndrome has variable definitions, abdominal obesity is an obligatory component; this provides rationale for leptin to be evaluated as a diagnostic tool [218]. The leptin-adiponectin ratio (LAR) has been confirmed to be a better diagnostic marker for metabolic syndrome than either hormone on its own, that is elevated leptin levels or decreased adiponectin levels [218]. Additionally, the LAR were more correlated with current diagnostic values such as body mass index, body adiposity, and waist circumference in comparison to another marker for dysfunctional adipose, the visceral adiposity index [219]. A standardized reference range has yet to be set for LAR. Frühbeck et al. set a value that accurately accounted for cardiometabolic risk; patients with obesity, type II diabetes mellitus, and metabolic syndrome all had leptin-adiponectin relationships that met the criteria for increased risk [219]. Although the LAR is not ordinarily used, it may serve as an estimator that can potentially account for a larger number of identified subjects at risk than just considering leptin alone.

### 13.2. Therapeutic Tool

The form of leptin that is currently available for human therapy is recombinant methionyl leptin, or metreleptin. It has been approved by the Food and Drug Administration to treat congenital or acquired lipodystrophy, with the purpose of normalizing blood lipids [220]. The drug aims to reduce triglycerides and increase HDL and has been successful for leptin-deficient adults [220,221]. Congenital leptin deficiency is very rare, but leptin replacement therapy has been shown to also decrease body weight, total fat mass, food intake, and plasma insulin for this small cohort of individuals [221]. Additionally, leptin replacement therapy is being evaluated as a viable treatment option for hypothalamic amenorrhea. In these patients, their state of energy deprivation is characterized by reduced fat mass and thus serum leptin concentration. Exogenous leptin was found to resolve anovulation and normalize thyroid, adrenal, and gonadal axes in multiple drug trials [222,223]. For these reasons, leptin is a promising therapeutic agent in the realm of women’s health.

It is worth mentioning the efforts being made to find a use for leptin in the treatment of diabetes. Regarding type I diabetes, leptin administration was found to improve blood sugar levels, increase glucose uptake, and modulate the autoimmune destruction of pancreatic beta cells. Although persuasive, these findings were true for animal models but have yet to be replicated in clinical trials [224]. Conversely, clinical trials have been underway for type II diabetes. Therapeutic leptin did not elicit significant changes in body weight, body composition, or insulin sensitivity [225]. One must consider leptin resistance in these participants, as individuals were mostly overweight and obese. Perhaps recombinant leptin in non-obese individuals with type II diabetes would have a different outcomes, but future research is needed to confirm or deny these speculations.

## 14. Conclusions

**Figure 1:**
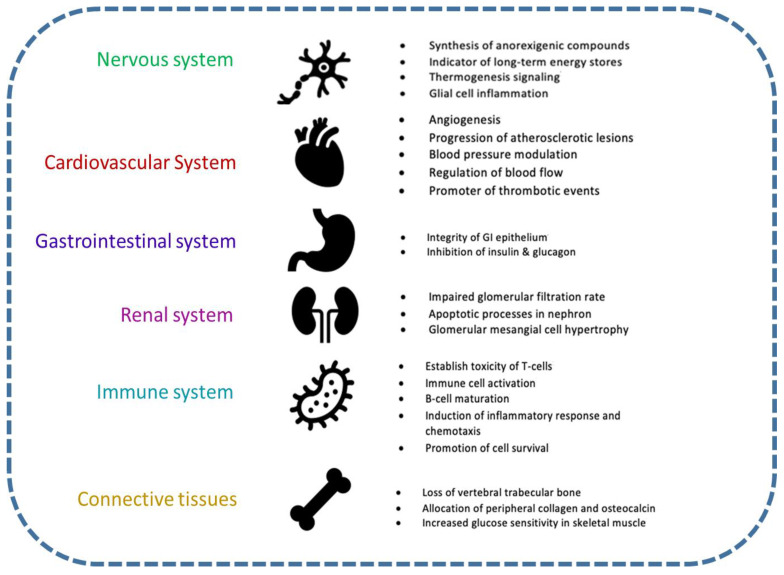
An illustration of the broad effects of leptin signaling within the human body. The role of leptin as an anorexic agent is well known. The role of leptin in regulating various physiological systems under normal and pathological conditions are explained here, including cardiovascular, gastro-intestinal, renal, immune, and skeletal systems.

To date, the influence of leptin and leptin receptor expression and regulation has been centered around obesity. Given that plasma leptin concentration is directly correlated to elevated body mass index and fat mass, it is rational to categorize leptin only as a weight-regulating peptide hormone [226]. In reality, the peripheral expression of leptin and its receptor may warrant extra obesogenic and anorexic effects but requiring thorough investigation [106,207,208]. The comorbidities associated with elevated fat mass may be partially explained from the peripheral pleiotropic effects of leptin [106,207,208]. The purpose of this review is to remove the fixation of leptin just as a target for only obesity research and instead to consider leptin as a connecting signal across multiple organ systems regulating metabolism, inflammation, and systemic homeostasis (Summary diagram–Figure 1). Our review portrays the diverse function of this peptide hormone in different organ systems. This review also reveals a gender-specific role for leptin with pronounced effects in females especially in the pre-menopausal stage or during the active reproductive cycle in comparison to age-matched male counterparts. Even though leptin levels can be considered as a systemic marker for obesity and metabolic syndrome in both genders, it is considered a more reliable diagnostic marker for different types of cancers in females.

As a clinical recommendation, leptin levels can be considered as an ideal diagnostic tool and marker for insulin resistance and metabolic syndrome in both genders, but leptin levels could serve as an appropriate marker for detecting cancer in females. Considering the high basal levels of serum levels in women during the pre-menopausal stage, it could play a regulatory role in systemic metabolic and endocrine functions. Thus, maintaining appropriate leptin levels in females could be quite crucial for their metabolic and systemic health. Many of the actions of leptin from head to toe in both genders are still unclear. Integrating information from basic and clinical studies should help us in revealing the unknown systemic role of this peptide hormone, both as a biomarker and as a therapeutic target.

## References

[1] Zhang F., Chen Y., Heiman M., Dimarchi R. (2005). Leptin: Structure, function and biology. Vitam. Horm..

[2] Kennedy G.C. (1953). The role of depot fat in the hypothalamic control of food intake in the rat. Proc. R. Soc. Lond. B Biol. Sci..

[3] Kelesidis T., Kelesidis I., Chou S., Mantzoros C.S. (2010). Narrative review: The role of leptin in human physiology: Emerging clinical applications. Ann. Intern. Med..

[4] Sahu A., Nguyen L., O’Doherty R.M. (2002). Nutritional regulation of hypothalamic leptin receptor gene expression is defective in diet-induced obesity. J. Neuroendocrinol..

[5] Sinha M.K., Caro J.F. (1998). Clinical aspects of leptin. Vitam. Horm..

[6] Hidaka S., Yoshimatsu H., Kondou S., Oka K., Tsuruta Y., Sakino H., Itateyama E., Noguchi H., Himeno K., Okamoto K. (2001). Hypoleptinemia, but not hypoinsulinemia, induces hyperphagia in streptozotocin-induced diabetic rats. J. Neurochem..

[7] Hileman S.M., Tornoe J., Flier J.S., Bjorbaek C. (2000). Transcellular transport of leptin by the short leptin receptor isoform ObRa in Madin-Darby Canine Kidney cells. Endocrinology.

[8] Morgan P.J., Ross A.W., Mercer J.G., Barrett P. (2006). What can we learn from seasonal animals about the regulation of energy balance?. Prog. Brain Res..

[9] Morton G.J., Gelling R.W., Niswender K.D., Morrison C.D., Rhodes C.J., Schwartz M.W. (2005). Leptin regulates insulin sensitivity via phosphatidylinositol-3-OH kinase signaling in mediobasal hypothalamic neurons. Cell Metab..

[10] Pal R., Sahu A. (2003). Leptin signaling in the hypothalamus during chronic central leptin infusion. Endocrinology.

[11] (2001). Sone M, Osamura RY: Leptin and the pituitary. Pituitary.

[12] Tsumanuma I., Jin L., Zhang S., Bayliss J.M., Scheithauer B.W., Lloyd R.V. (2000). Leptin signal transduction in the HP75 human pituitary cell line. Pituitary.

[13] Bjorbaek C., Uotani S., Da Silva B., Flier J.S. (1997). Divergent signaling capacities of the long and short isoforms of the leptin receptor. J. Biol. Chem..

[14] Da Silva B.A., Bjorbaek C., Uotani S., Flier J.S. (1998). Functional properties of leptin receptor isoforms containing the gln-->pro extracellular domain mutation of the fatty rat. Endocrinology.

[15] Sanchez-Margalet V., Martin-Romero C., Gonzalez-Yanes C., Goberna R., Rodriguez-Bano J., Muniain M.A. (2002). Leptin receptor (Ob-R) expression is induced in peripheral blood mononuclear cells by in vitro activation and in vivo in HIV-infected patients. Clin. Exp. Immunol..

[16] Gorska E., Popko K., Stelmaszczyk-Emmel A., Ciepiela O., Kucharska A., Wasik M. (2010). Leptin receptors. Eur. J. Med. Res..

[17] Hakansson-Ovesjo M.L., Collin M., Meister B. (2000). Down-regulated STAT3 messenger ribonucleic acid and STAT3 protein in the hypothalamic arcuate nucleus of the obese leptin-deficient (ob/ob) mouse. Endocrinology.

[18] Kowalski T.J., Liu S.M., Leibel R.L., Chua S.C. (2001). Transgenic complementation of leptin-receptor deficiency. I. Rescue of the obesity/diabetes phenotype of LEPR-null mice expressing a LEPR-B transgene. Diabetes.

[19] Fruhbeck G. (2006). Intracellular signalling pathways activated by leptin. Biochem. J..

[20] Heshka J.T., Jones P.J. (2001). A role for dietary fat in leptin receptor, OB-Rb, function. Life Sci..

[21] Meister B. (2000). Control of food intake via leptin receptors in the hypothalamus. Vitam. Horm..

[22] Ovesjo M.L., Gamstedt M., Collin M., Meister B. (2001). GABAergic nature of hypothalamic leptin target neurones in the ventromedial arcuate nucleus. J. Neuroendocrinol..

[23] Chen P., Williams S.M., Grove K.L., Smith M.S. (2004). Melanocortin 4 receptor-mediated hyperphagia and activation of neuropeptide Y expression in the dorsomedial hypothalamus during lactation. J. Neurosci..

[24] De Luca C., Kowalski T.J., Zhang Y., Elmquist J.K., Lee C., Kilimann M.W., Ludwig T., Liu S.M., Chua S.C. (2005). Complete rescue of obesity, diabetes, and infertility in db/db mice by neuron-specific LEPR-B transgenes. J. Clin. Investig..

[25] Kaneda T., Makino S., Nishiyama M., Asaba K., Hashimoto K. (2001). Differential neuropeptide responses to starvation with ageing. J. Neuroendocrinol..

[26] Lonnerdal B., Havel P.J. (2000). Serum leptin concentrations in infants: Effects of diet, sex, and adiposity. Am. J. Clin. Nutr..

[27] Cooper J.A., Polonsky K.S., Schoeller D.A. (2009). Serum leptin levels in obese males during over- and underfeeding. Obesity.

[28] Adamska-Patruno E., Ostrowska L., Goscik J., Pietraszewska B., Kretowski A., Gorska M. (2018). The relationship between the leptin/ghrelin ratio and meals with various macronutrient contents in men with different nutritional status: A randomized crossover study. Nutr. J..

[29] Muoio D.M., Dohm G.L. (2002). Peripheral metabolic actions of leptin. Best Pract. Res. Clin. Endocrinol. Metab..

[30] Aparicio T., Kermorgant S., Darmoul D., Guilmeau S., Hormi K., Mahieu-Caputo D., Lehy T. (2005). Leptin and Ob-Rb receptor isoform in the human digestive tract during fetal development. J. Clin. Endocrinol. Metab..

[31] Breidert M., Miehlke S., Glasow A., Orban Z., Stolte M., Ehninger G., Bayerdorffer E., Nettesheim O., Halm U., Haidan A. (1999). Leptin and its receptor in normal human gastric mucosa and in Helicobacter pylori-associated gastritis. Scand. J. Gastroenterol..

[32] Mix H., Widjaja A., Jandl O., Cornberg M., Kaul A., Goke M., Beil W., Kuske M., Brabant G., Manns M.P. (2000). Expression of leptin and leptin receptor isoforms in the human stomach. Gut.

[33] Arita S., Inagaki-Ohara K. (2019). High-fat-diet-induced modulations of leptin signaling and gastric microbiota drive precancerous lesions in the stomach. Nutrition.

[34] Merigo F., Brandolese A., Facchin S., Boschi F., Di Chio M., Savarino E., D’Inca R., Sturniolo G.C., Sbarbati A. (2021). Immunolocalization of leptin and leptin receptor in colorectal mucosa of ulcerative colitis, Crohn’s disease and control subjects with no inflammatory bowel disease. Cell Tissue Res..

[35] Tian Y.M., Tian S.Y., Wang D., Cui F., Zhang X.J., Zhang Y. (2019). Elevated expression of the leptin receptor obR may contribute to inflammation in patients with ulcerative colitis. Mol. Med. Rep..

[36] Islam M.S., Morton N.M., Hansson A., Emilsson V. (1997). Rat insulinoma-derived pancreatic beta-cells express a functional leptin receptor that mediates a proliferative response. Biochem. Biophys. Res. Commun..

[37] Xu Y., Tan M., Tian X., Zhang J., Zhang J., Chen J., Xu W., Sheng H. (2020). Leptin receptor mediates the proliferation and glucose metabolism of pancreatic cancer cells via AKT pathway activation. Mol. Med. Rep..

[38] Yuan L., An H., Deng X., Li Z. (2003). Regulation of leptin on insulin secretion and sulfonulurea receptor 1 transcription level in isolated rats pancreatic islets. Chin. Med. J..

[39] Emilsson V., Liu Y.L., Cawthorne M.A., Morton N.M., Davenport M. (1997). Expression of the functional leptin receptor mRNA in pancreatic islets and direct inhibitory action of leptin on insulin secretion. Diabetes.

[40] Pereira S., Cline D.L., Glavas M.M., Covey S.D., Kieffer T.J. (2021). Tissue-Specific Effects of Leptin on Glucose and Lipid Metabolism. Endocr. Rev..

[41] Kieffer T.J., Heller R.S., Leech C.A., Holz G.G., Habener J.F. (1997). Leptin suppression of insulin secretion by the activation of ATP-sensitive K+ channels in pancreatic beta-cells. Diabetes.

[42] Fu Z., Gilbert E.R., Liu D. (2013). Regulation of insulin synthesis and secretion and pancreatic Beta-cell dysfunction in diabetes. Curr. Diabetes Rev..

[43] Cases J.A., Gabriely I., Ma X.H., Yang X.M., Michaeli T., Fleischer N., Rossetti L., Barzilai N. (2001). Physiological increase in plasma leptin markedly inhibits insulin secretion in vivo. Diabetes.

[44] Cong L., Chen K., Li J., Gao P., Li Q., Mi S., Wu X., Zhao A.Z. (2007). Regulation of adiponectin and leptin secretion and expression by insulin through a PI3K-PDE3B dependent mechanism in rat primary adipocytes. Biochem. J..

[45] Martinez-Una M., Lopez-Mancheno Y., Dieguez C., Fernandez-Rojo M.A., Novelle M.G. (2020). Unraveling the Role of Leptin in Liver Function and Its Relationship with Liver Diseases. Int. J. Mol. Sci..

[46] Huynh F.K., Levi J., Denroche H.C., Gray S.L., Voshol P.J., Neumann U.H., Speck M., Chua S.C., Covey S.D., Kieffer T.J. (2010). Disruption of hepatic leptin signaling protects mice from age- and diet-related glucose intolerance. Diabetes.

[47] Yang S.Q., Lin H.Z., Mandal A.K., Huang J., Diehl A.M. (2001). Disrupted signaling and inhibited regeneration in obese mice with fatty livers: Implications for nonalcoholic fatty liver disease pathophysiology. Hepatology.

[48] Leclercq I.A., Farrell G.C., Schriemer R., Robertson G.R. (2002). Leptin is essential for the hepatic fibrogenic response to chronic liver injury. J. Hepatol..

[49] Polyzos S.A., Aronis K.N., Kountouras J., Raptis D.D., Vasiloglou M.F., Mantzoros C.S. (2016). Circulating leptin in non-alcoholic fatty liver disease: A systematic review and meta-analysis. Diabetologia.

[50] Ducy P., Amling M., Takeda S., Priemel M., Schilling A.F., Beil F.T., Shen J., Vinson C., Rueger J.M., Karsenty G. (2000). Leptin inhibits bone formation through a hypothalamic relay: A central control of bone mass. Cell.

[51] Pogoda P., Egermann M., Schnell J.C., Priemel M., Schilling A.F., Alini M., Schinke T., Rueger J.M., Schneider E., Clarke I. (2006). Leptin inhibits bone formation not only in rodents, but also in sheep. J. Bone Miner. Res..

[52] Iwaniec U.T., Boghossian S., Trevisiol C.H., Wronski T.J., Turner R.T., Kalra S.P. (2011). Hypothalamic leptin gene therapy prevents weight gain without long-term detrimental effects on bone in growing and skeletally mature female rats. J. Bone Miner. Res..

[53] De Blasio M.J., Lanham S.A., Blache D., Oreffo R.O.C., Fowden A.L., Forhead A.J. (2018). Sex- and bone-specific responses in bone structure to exogenous leptin and leptin receptor antagonism in the ovine fetus. Am. J. Physiol. Regul. Integr. Comp. Physiol..

[54] Han Y.C., Ma B., Guo S., Yang M., Li L.J., Wang S.J., Tan J. (2018). Leptin regulates disc cartilage endplate degeneration and ossification through activation of the MAPK-ERK signalling pathway in vivo and in vitro. J. Cell. Mol. Med..

[55] Reid I.R., Baldock P.A., Cornish J. (2018). Effects of Leptin on the Skeleton. Endocr. Rev..

[56] Gordeladze J.O., Drevon C.A., Syversen U., Reseland J.E. (2002). Leptin stimulates human osteoblastic cell proliferation, de novo collagen synthesis, and mineralization: Impact on differentiation markers, apoptosis, and osteoclastic signaling. J. Cell. Biochem..

[57] Hui W., Litherland G.J., Elias M.S., Kitson G.I., Cawston T.E., Rowan A.D., Young D.A. (2012). Leptin produced by joint white adipose tissue induces cartilage degradation via upregulation and activation of matrix metalloproteinases. Ann. Rheum. Dis..

[58] Burguera B., Hofbauer L.C., Thomas T., Gori F., Evans G.L., Khosla S., Riggs B.L., Turner R.T. (2001). Leptin reduces ovariectomy-induced bone loss in rats. Endocrinology.

[59] Upadhyay J., Farr O.M., Mantzoros C.S. (2015). The role of leptin in regulating bone metabolism. Metabolism.

[60] Papathanassoglou E., El-Haschimi K., Li X.C., Matarese G., Strom T., Mantzoros C. (2006). Leptin receptor expression and signaling in lymphocytes: Kinetics during lymphocyte activation, role in lymphocyte survival, and response to high fat diet in mice. J. Immunol..

[61] Saucillo D.C., Gerriets V.A., Sheng J., Rathmell J.C., Maciver N.J. (2014). Leptin metabolically licenses T cells for activation to link nutrition and immunity. J. Immunol..

[62] Fernandez-Riejos P., Goberna R., Sanchez-Margalet V. (2008). Leptin promotes cell survival and activates Jurkat T lymphocytes by stimulation of mitogen-activated protein kinase. Clin. Exp. Immunol..

[63] Martin-Romero C., Santos-Alvarez J., Goberna R., Sanchez-Margalet V. (2000). Human leptin enhances activation and proliferation of human circulating T lymphocytes. Cell. Immunol..

[64] Matarese G., Carrieri P.B., La Cava A., Perna F., Sanna V., De Rosa V., Aufiero D., Fontana S., Zappacosta S. (2005). Leptin increase in multiple sclerosis associates with reduced number of CD4(+)CD25+ regulatory T cells. Proc. Natl. Acad. Sci. USA.

[65] Mattioli B., Straface E., Quaranta M.G., Giordani L., Viora M. (2005). Leptin promotes differentiation and survival of human dendritic cells and licenses them for Th1 priming. J. Immunol..

[66] Galan-Diez M., Cuesta-Dominguez A., Kousteni S. (2018). The Bone Marrow Microenvironment in Health and Myeloid Malignancy. Cold Spring Harb. Perspect. Med..

[67] Gorska E., Popko K., Wasik M. (2013). Leptin receptor in childhood acute leukemias. Adv. Exp. Med. Biol..

[68] Han T.J., Wang X. (2015). Leptin and its receptor in hematologic malignancies. Int. J. Clin. Exp. Med..

[69] Konopleva M., Mikhail A., Estrov Z., Zhao S., Harris D., Sanchez-Williams G., Kornblau S.M., Dong J., Kliche K.O., Jiang S. (1999). Expression and function of leptin receptor isoforms in myeloid leukemia and myelodysplastic syndromes: Proliferative and anti-apoptotic activities. Blood.

[70] Morris E.V., Edwards C.M. (2018). Adipokines, adiposity, and bone marrow adipocytes: Dangerous accomplices in multiple myeloma. J. Cell. Physiol..

[71] Diaz-Blanco E., Bruns I., Neumann F., Fischer J.C., Graef T., Rosskopf M., Brors B., Pechtel S., Bork S., Koch A. (2007). Molecular signature of CD34(+) hematopoietic stem and progenitor cells of patients with CML in chronic phase. Leukemia.

[72] Sadim M., Xu Y., Selig K., Paulus J., Uthe R., Agarwl S., Dubin I., Oikonomopoulou P., Zaichenko L., McCandlish S.A. (2017). A prospective evaluation of clinical and genetic predictors of weight changes in breast cancer survivors. Cancer.

[73] Nakao T., Hino M., Yamane T., Nishizawa Y., Morii H., Tatsumi N. (1998). Expression of the leptin receptor in human leukaemic blast cells. Br. J. Haematol..

[74] Keshavarz H., Meints L.M., Geiger M.K., Zinn K.R., Spence D.M. (2021). Specific Binding of Leptin to Red Blood Cells Delivers a Pancreatic Hormone and Stimulates ATP Release. Mol. Pharm..

[75] Cabrera de Leon A., Gonzalez D.A., Mendez L.I., Aguirre-Jaime A., Del Cristo Rodriguez Perez M., Coello S.D., Trujillo I.C. (2004). Leptin and altitude in the cardiovascular diseases. Obes. Res..

[76] Hou N., Luo J.D. (2011). Leptin and cardiovascular diseases. Clin. Exp. Pharmacol. Physiol..

[77] Katsiki N., Mikhailidis D.P., Banach M. (2018). Leptin, cardiovascular diseases and type 2 diabetes mellitus. Acta Pharmacol. Sin..

[78] Luo J.D., Zhang G.S., Chen M.S. (2005). Leptin and cardiovascular diseases. Timely Top. Med. Cardiovasc. Dis..

[79] Peelman F., Waelput W., Iserentant H., Lavens D., Eyckerman S., Zabeau L., Tavernier J. (2004). Leptin: Linking adipocyte metabolism with cardiovascular and autoimmune diseases. Prog. Lipid Res..

[80] Mikhail A.A., Beck E.X., Shafer A., Barut B., Gbur J.S., Zupancic T.J., Schweitzer A.C., Cioffi J.A., Lacaud G., Ouyang B. (1997). Leptin stimulates fetal and adult erythroid and myeloid development. Blood.

[81] Sivan E., Lin W.M., Homko C.J., Reece E.A., Boden G. (1997). Leptin is present in human cord blood. Diabetes.

[82] Trinh T., Broxmeyer H.E. (2021). Role for Leptin and Leptin Receptors in Stem Cells During Health and Diseases. Stem Cell Rev. Rep..

[83] Tsiotra P.C., Pappa V., Raptis S.A., Tsigos C. (2000). Expression of the long and short leptin receptor isoforms in peripheral blood mononuclear cells: Implications for leptin’s actions. Metabolism.

[84] Wolk R., Deb A., Caplice N.M., Somers V.K. (2005). Leptin receptor and functional effects of leptin in human endothelial progenitor cells. Atherosclerosis.

[85] Corsonello A., Perticone F., Malara A., De Domenico D., Loddo S., Buemi M., Ientile R., Corica F. (2003). Leptin-dependent platelet aggregation in healthy, overweight and obese subjects. Int. J. Obes. Relat. Metab. Disord..

[86] Elbatarny H.S., Maurice D.H. (2005). Leptin-mediated activation of human platelets: Involvement of a leptin receptor and phosphodiesterase 3A-containing cellular signaling complex. Am. J. Physiol. Endocrinol. Metab..

[87] Konstantinides S., Schafer K., Koschnick S., Loskutoff D.J. (2001). Leptin-dependent platelet aggregation and arterial thrombosis suggests a mechanism for atherothrombotic disease in obesity. J. Clin. Investig..

[88] Konstantinides S., Schafer K., Loskutoff D.J. (2001). The prothrombotic effects of leptin possible implications for the risk of cardiovascular disease in obesity. Ann. N. Y. Acad. Sci..

[89] Nakata M., Yada T., Soejima N., Maruyama I. (1999). Leptin promotes aggregation of human platelets via the long form of its receptor. Diabetes.

[90] Frank S., Stallmeyer B., Kampfer H., Kolb N., Pfeilschifter J. (2000). Leptin enhances wound re-epithelialization and constitutes a direct function of leptin in skin repair. J. Clin. Investig..

[91] Murad A., Nath A.K., Cha S.T., Demir E., Flores-Riveros J., Sierra-Honigmann M.R. (2003). Leptin is an autocrine/paracrine regulator of wound healing. FASEB J..

[92] Schafer K., Halle M., Goeschen C., Dellas C., Pynn M., Loskutoff D.J., Konstantinides S. (2004). Leptin promotes vascular remodeling and neointimal growth in mice. Arterioscler. Thromb. Vasc. Biol..

[93] Tadokoro S., Ide S., Tokuyama R., Umeki H., Tatehara S., Kataoka S., Satomura K. (2015). Leptin promotes wound healing in the skin. PLoS ONE.

[94] Umeki H., Tokuyama R., Ide S., Okubo M., Tadokoro S., Tezuka M., Tatehara S., Satomura K. (2014). Leptin promotes wound healing in the oral mucosa. PLoS ONE.

[95] Beltowski J. (2006). Leptin and atherosclerosis. Atherosclerosis.

[96] Bouloumie A., Drexler H.C., Lafontan M., Busse R. (1998). Leptin, the product of Ob gene, promotes angiogenesis. Circ. Res..

[97] Garonna E., Botham K.M., Birdsey G.M., Randi A.M., Gonzalez-Perez R.R., Wheeler-Jones C.P. (2011). Vascular endothelial growth factor receptor-2 couples cyclo-oxygenase-2 with pro-angiogenic actions of leptin on human endothelial cells. PLoS ONE.

[98] Heida N.M., Leifheit-Nestler M., Schroeter M.R., Muller J.P., Cheng I.F., Henkel S., Limbourg A., Limbourg F.P., Alves F., Quigley J.P. (2010). Leptin enhances the potency of circulating angiogenic cells via src kinase and integrin (alpha)vbeta5: Implications for angiogenesis in human obesity. Arterioscler. Thromb. Vasc. Biol..

[99] Park H.Y., Kwon H.M., Lim H.J., Hong B.K., Lee J.Y., Park B.E., Jang Y., Cho S.Y., Kim H.S. (2001). Potential role of leptin in angiogenesis: Leptin induces endothelial cell proliferation and expression of matrix metalloproteinases in vivo and in vitro. Exp. Mol. Med..

[100] Kang S.M., Kwon H.M., Hong B.K., Kim D., Kim I.J., Choi E.Y., Jang Y., Kim H.S., Kim M.S., Kwon H.C. (2000). Expression of leptin receptor (Ob-R) in human atherosclerotic lesions: Potential role in intimal neovascularization. Yonsei Med. J..

[101] Hall J.E., Do Carmo J.M., Da Silva A.A., Wang Z., Hall M.E. (2019). Obesity, kidney dysfunction and hypertension: Mechanistic links. Nat. Rev. Nephrol..

[102] Vilahur G., Ben-Aicha S., Badimon L. (2017). New insights into the role of adipose tissue in thrombosis. Cardiovasc. Res..

[103] Xue B., Yu Y., Beltz T.G., Guo F., Felder R.B., Wei S.G., Kim Johnson A. (2021). Maternal Angiotensin II-Induced Hypertension Sensitizes Postweaning High-Fat Diet-Elicited Hypertensive Response Through Increased Brain Reactivity in Rat Offspring. J. Am. Heart Assoc..

[104] Xue B., Yu Y., Zhang Z., Guo F., Beltz T.G., Thunhorst R.L., Felder R.B., Johnson A.K. (2016). Leptin Mediates High-Fat Diet Sensitization of Angiotensin II-Elicited Hypertension by Upregulating the Brain Renin-Angiotensin System and Inflammation. Hypertension.

[105] Da Fonseca A.C.P., Abreu G.M., Zembrzuski V.M., Campos Junior M., Carneiro J.R.I., Nogueira Neto J.F., Magno F., Rosado E.L., Bozza P.T., De Cabello G.M.K. (2021). Study of LEP, MRAP2 and POMC genes as potential causes of severe obesity in Brazilian patients. Eat. Weight Disord..

[106] Yoo S.B., Ryu V., Park E.Y., Kim B.T., Kang D.W., Lee J.H., Jahng J.W. (2011). The arcuate NPY, POMC, and CART expressions responding to food deprivation are exaggerated in young female rats that experienced neonatal maternal separation. Neuropeptides.

[107] Elias C.F., Lee C., Kelly J., Aschkenasi C., Ahima R.S., Couceyro P.R., Kuhar M.J., Saper C.B., Elmquist J.K. (1998). Leptin activates hypothalamic CART neurons projecting to the spinal cord. Neuron.

[108] Khokhar K.K., Sidhu S., Kaur G. (2010). Correlation between leptin level and hypertension in normal and obese pre- and postmenopausal women. Eur. J. Endocrinol..

[109] Smith C.C., Mocanu M.M., Davidson S.M., Wynne A.M., Simpkin J.C., Yellon D.M. (2006). Leptin, the obesity-associated hormone, exhibits direct cardioprotective effects. Br. J. Pharmacol..

[110] Kamimura D., Suzuki T., Wang W., DeShazo M., Hall J.E., Winniford M.D., Kullo I.J., Mosley T.H., Butler K.R., Hall M.E. (2018). Higher plasma leptin levels are associated with reduced left ventricular mass and left ventricular diastolic stiffness in black women: Insights from the Genetic Epidemiology Network of Arteriopathy (GENOA) study. Hypertens. Res..

[111] Korczynska J., Czumaj A., Chmielewski M., Swierczynski J., Sledzinski T. (2021). The Causes and Potential Injurious Effects of Elevated Serum Leptin Levels in Chronic Kidney Disease Patients. Int. J. Mol. Sci..

[112] Iida M., Murakami T., Yamada M., Sei M., Kuwajima M., Mizuno A., Noma Y., Aono T., Shima K. (1996). Hyperleptinemia in chronic renal failure. Horm. Metab. Res..

[113] Mak R.H., Cheung W., Cone R.D., Marks D.L. (2006). Leptin and inflammation-associated cachexia in chronic kidney disease. Kidney Int..

[114] Stenvinkel P. (1999). Leptin and its clinical implications in chronic renal failure. Miner. Electrolyte Metab..

[115] Alix P.M., Guebre-Egziabher F., Soulage C.O. (2014). Leptin as an uremic toxin: Deleterious role of leptin in chronic kidney disease. Biochimie.

[116] Johansen J.E., Broberger C., Lavebratt C., Johansson C., Kuhar M.J., Hokfelt T., Schalling M. (2000). Hypothalamic CART and serum leptin levels are reduced in the anorectic (anx/anx) mouse. Brain Res. Mol. Brain Res..

[117] Senn S.S., Le Foll C., Whiting L., Tarasco E., Duffy S., Lutz T.A., Boyle C.N. (2019). Unsilencing of native LepRs in hypothalamic SF1 neurons does not rescue obese phenotype in LepR-deficient mice. Am. J. Physiol. Regul. Integr. Comp. Physiol..

[118] Cardinal P., Andre C., Quarta C., Bellocchio L., Clark S., Elie M., Leste-Lasserre T., Maitre M., Gonzales D., Cannich A. (2014). CB1 cannabinoid receptor in SF1-expressing neurons of the ventromedial hypothalamus determines metabolic responses to diet and leptin. Mol. Metab..

[119] Valdearcos M., Xu A.W., Koliwad S.K. (2015). Hypothalamic inflammation in the control of metabolic function. Annu. Rev. Physiol..

[120] Le Foll C., Johnson M.D., Dunn-Meynell A.A., Boyle C.N., Lutz T.A., Levin B.E. (2015). Amylin-induced central IL-6 production enhances ventromedial hypothalamic leptin signaling. Diabetes.

[121] Kim C.K., Ryu W.S., Choi I.Y., Kim Y.J., Rim D., Kim B.J., Jang H., Yoon B.W., Lee S.H. (2013). Detrimental effects of leptin on intracerebral hemorrhage via the STAT3 signal pathway. J. Cereb. Blood Flow Metab..

[122] Guerrero-Garcia J.J., Carrera-Quintanar L., Lopez-Roa R.I., Marquez-Aguirre A.L., Rojas-Mayorquin A.E., Ortuno-Sahagun D. (2016). Multiple Sclerosis and Obesity: Possible Roles of Adipokines. Mediat. Inflamm..

[123] Fujita Y., Yamashita T. (2019). The Effects of Leptin on Glial Cells in Neurological Diseases. Front. Neurosci..

[124] De Git K.C., Adan R.A. (2015). Leptin resistance in diet-induced obesity: The role of hypothalamic inflammation. Obes. Rev..

[125] Garcia-Estevez L., Gonzalez-Martinez S., Moreno-Bueno G. (2021). The Leptin Axis and Its Association with the Adaptive Immune System in Breast Cancer. Front. Immunol..

[126] De Leon-Guerrero S.D., Salazar-Leon J., Meza-Sosa K.F., Valle-Garcia D., Aguilar-Leon D., Pedraza-Alva G., Perez-Martinez L. (2022). An enriched environment reestablishes metabolic homeostasis by reducing obesity-induced inflammation. Dis. Models Mech..

[127] Al-Hussaniy H.A., Alburghaif A.H., Naji M.A. (2021). Leptin hormone and its effectiveness in reproduction, metabolism, immunity, diabetes, hopes and ambitions. J. Med. Life.

[128] Sanchez-Garrido J., Shenoy A.R. (2021). Regulation and repurposing of nutrient sensing and autophagy in innate immunity. Autophagy.

[129] Richer B.C., Salei N., Laskay T., Seeger K. (2018). Changes in Neutrophil Metabolism upon Activation and Aging. Inflammation.

[130] Lovaszi M., Haas C.B., Antonioli L., Pacher P., Hasko G. (2021). The role of P2Y receptors in regulating immunity and metabolism. Biochem. Pharmacol..

[131] Adinolfi E., Giuliani A.L., De Marchi E., Pegoraro A., Orioli E., Di Virgilio F. (2018). The P2X7 receptor: A main player in inflammation. Biochem. Pharmacol..

[132] Sennello J.A., Fayad R., Morris A.M., Eckel R.H., Asilmaz E., Montez J., Friedman J.M., Dinarello C.A., Fantuzzi G. (2005). Regulation of T cell-mediated hepatic inflammation by adiponectin and leptin. Endocrinology.

[133] Oswald J., Buttner M., Jasinski-Bergner S., Jacobs R., Rosenstock P., Kielstein H. (2018). Leptin affects filopodia and cofilin in NK-92 cells in a dose- and time-dependent manner. Eur. J. Histochem..

[134] Naylor C., Petri W.A. (2016). Leptin Regulation of Immune Responses. Trends Mol. Med..

[135] Lo C.K., Lam Q.L., Yang M., Ko K.H., Sun L., Ma R., Wang S., Xu H., Tam S., Wu C.Y. (2009). Leptin signaling protects NK cells from apoptosis during development in mouse bone marrow. Cell. Mol. Immunol..

[136] Lamas B., Goncalves-Mendes N., Nachat-Kappes R., Rossary A., Caldefie-Chezet F., Vasson M.P., Farges M.C. (2013). Leptin modulates dose-dependently the metabolic and cytolytic activities of NK-92 cells. J. Cell. Physiol..

[137] Han H., Zhou W. (2019). Leptin and Its Derivatives: A Potential Target for Autoimmune Diseases. Curr. Drug Targets.

[138] Grases-Pinto B., Abril-Gil M., Rodriguez-Lagunas M.J., Castell M., Perez-Cano F.J., Franch A. (2018). Leptin and adiponectin supplementation modifies mesenteric lymph node lymphocyte composition and functionality in suckling rats. Br. J. Nutr..

[139] Matarese G., Castelli-Gattinara G., Cancrini C., Bernardi S., Romiti M.L., Savarese C., Di Giacomo A., Rossi P., Racioppi L. (2002). Serum leptin and CD4+ T lymphocytes in HIV+ children during highly active antiretroviral therapy. Clin. Endocrinol..

[140] Matarese G. (2000). Leptin and the immune system: How nutritional status influences the immune response. Eur. Cytokine Netw..

[141] Lord G.M., Matarese G., Howard J.K., Baker R.J., Bloom S.R., Lechler R.I. (1998). Leptin modulates the T-cell immune response and reverses starvation-induced immunosuppression. Nature.

[142] Deng J., Liu Y., Yang M., Wang S., Zhang M., Wang X., Ko K.H., Hua Z., Sun L., Cao X. (2012). Leptin exacerbates collagen-induced arthritis via enhancement of Th17 cell response. Arthritis Rheum..

[143] Batra A., Okur B., Glauben R., Erben U., Ihbe J., Stroh T., Fedke I., Chang H.D., Zeitz M., Siegmund B. (2010). Leptin: A critical regulator of CD4+ T-cell polarization in vitro and in vivo. Endocrinology.

[144] Toro A.R., Maymo J.L., Ibarbalz F.M., Perez-Perez A., Maskin B., Faletti A.G., Sanchez-Margalet V., Varone C.L. (2014). Leptin is an anti-apoptotic effector in placental cells involving p53 downregulation. PLoS ONE.

[145] Ogunwobi O.O., Beales I.L. (2007). The anti-apoptotic and growth stimulatory actions of leptin in human colon cancer cells involves activation of JNK mitogen activated protein kinase, JAK2 and PI3 kinase/Akt. Int. J. Colorectal Dis..

[146] Da Silva S.V., Salama C., Renovato-Martins M., Helal-Neto E., Citelli M., Savino W., Barja-Fidalgo C. (2013). Increased leptin response and inhibition of apoptosis in thymocytes of young rats offspring from protein deprived dams during lactation. PLoS ONE.

[147] Rafique N., Salem A.M., Latif R., ALSheikh M.H. (2018). Serum leptin level across different phases of menstrual cycle in normal weight and overweight/obese females. Gynecol. Endocrinol..

[148] Zeng Q., Luo X., Han M., Liu W., Li H. (2018). Leptin/Osteopontin Axis Regulated Type 2T Helper Cell Response in Allergic Rhinitis with Obesity. EBioMedicine.

[149] Reis B.S., Lee K., Fanok M.H., Mascaraque C., Amoury M., Cohn L.B., Rogoz A., Dallner O.S., Moraes-Vieira P.M., Domingos A.I. (2015). Leptin receptor signaling in T cells is required for Th17 differentiation. J. Immunol..

[150] Cassano S., Pucino V., La Rocca C., Procaccini C., De Rosa V., Marone G., Matarese G. (2014). Leptin modulates autophagy in human CD4+CD25- conventional T cells. Metabolism.

[151] Sylvia K.E., Lorenz T.K., Heiman J.R., Demas G.E. (2018). Physiological predictors of leptin vary during menses and ovulation in healthy women. Reprod. Biol..

[152] Okudan N., Gokbel H., Ucok K., Baltaci A. (2005). Serum leptin concentration and anaerobic performance do not change during the menstrual cycle of young females. Neuroendocrinol. Lett..

[153] Morad V., Abrahamsson A., Dabrosin C. (2014). Estradiol affects extracellular leptin: Adiponectin ratio in human breast tissue in vivo. J. Clin. Endocrinol. Metab..

[154] Einollahi N., Dashti N., Nabatchian F. (2010). Serum leptin concentrations during the menstrual cycle in Iranian healthy women. Acta Med. Iran..

[155] Al-Harithy R.N., Al-Doghaither H., Abualnaja K. (2006). Correlation of leptin and sex hormones with endocrine changes in healthy Saudi women of different body weights. Ann. Saudi Med..

[156] Ahrens K., Mumford S.L., Schliep K.C., Kissell K.A., Perkins N.J., Wactawski-Wende J., Schisterman E.F. (2014). Serum leptin levels and reproductive function during the menstrual cycle. Am. J. Obstet. Gynecol..

[157] Sahu M., Sahu A. (2015). Leptin receptor expressing neurons express phosphodiesterase-3B (PDE3B) and leptin induces STAT3 activation in PDE3B neurons in the mouse hypothalamus. Peptides.

[158] Quennell J.H., Mulligan A.C., Tups A., Liu X., Phipps S.J., Kemp C.J., Herbison A.E., Grattan D.R., Anderson G.M. (2009). Leptin indirectly regulates gonadotropin-releasing hormone neuronal function. Endocrinology.

[159] Del Bianco-Borges B., Franci C.R. (2015). Estrogen-dependent post-translational change in the nitric oxide system may mediate the leptin action on LH and prolactin secretion. Brain Res..

[160] Yin N., Wang D., Zhang H., Yi X., Sun X., Shi B., Wu H., Wu G., Wang X., Shang Y. (2004). Molecular mechanisms involved in the growth stimulation of breast cancer cells by leptin. Cancer Res..

[161] Ishikawa M., Kitayama J., Nagawa H. (2004). Enhanced expression of leptin and leptin receptor (OB-R) in human breast cancer. Clin. Cancer Res..

[162] Garofalo C., Koda M., Cascio S., Sulkowska M., Kanczuga-Koda L., Golaszewska J., Russo A., Sulkowski S., Surmacz E. (2006). Increased expression of leptin and the leptin receptor as a marker of breast cancer progression: Possible role of obesity-related stimuli. Clin. Cancer Res..

[163] Sundaram S., Yan L. (2016). High-fat Diet Enhances Mammary Tumorigenesis and Pulmonary Metastasis and Alters Inflammatory and Angiogenic Profiles in MMTV-PyMT Mice. Anticancer Res..

[164] Garcia M.R., Steinbauer B., Srivastava K., Singhal M., Mattijssen F., Maida A., Christian S., Hess-Stumpp H., Augustin H.G., Muller-Decker K. (2017). Acetyl-CoA Carboxylase 1-Dependent Protein Acetylation Controls Breast Cancer Metastasis and Recurrence. Cell Metab..

[165] Landry D.A., Sormany F., Hache J., Roumaud P., Martin L.J. (2017). Steroidogenic genes expressions are repressed by high levels of leptin and the JAK/STAT signaling pathway in MA-10 Leydig cells. Mol. Cell. Biochem..

[166] Herrid M., O’Shea T., McFarlane J.R. (2008). Ontogeny of leptin and its receptor expression in mouse testis during the postnatal period. Mol. Reprod. Dev..

[167] Giovambattista A., Suescun M.O., Nessralla C.C., Franca L.R., Spinedi E., Calandra R.S. (2003). Modulatory effects of leptin on leydig cell function of normal and hyperleptinemic rats. Neuroendocrinology.

[168] Caprio M., Isidori A.M., Carta A.R., Moretti C., Dufau M.L., Fabbri A. (1999). Expression of functional leptin receptors in rodent Leydig cells. Endocrinology.

[169] Zhang J., Jin P.P., Gong M., Yi Q.T., Zhu R.J. (2018). Role of leptin and the leptin receptor in the pathogenesis of varicocele-induced testicular dysfunction. Mol. Med. Rep..

[170] Iyengar N.M., Brown K.A., Zhou X.K., Gucalp A., Subbaramaiah K., Giri D.D., Zahid H., Bhardwaj P., Wendel N.K., Falcone D.J. (2017). Metabolic Obesity, Adipose Inflammation and Elevated Breast Aromatase in Women with Normal Body Mass Index. Cancer Prev. Res..

[171] Alshaker H., Wang Q., Frampton A.E., Krell J., Waxman J., Winkler M., Stebbing J., Cooper C., Yague E., Pchejetski D. (2015). Sphingosine kinase 1 contributes to leptin-induced STAT3 phosphorylation through IL-6/gp130 transactivation in oestrogen receptor-negative breast cancer. Breast Cancer Res. Treat..

[172] Alshaker H., Krell J., Frampton A.E., Waxman J., Blyuss O., Zaikin A., Winkler M., Stebbing J., Yague E., Pchejetski D. (2014). Leptin induces upregulation of sphingosine kinase 1 in oestrogen receptor-negative breast cancer via Src family kinase-mediated, janus kinase 2-independent pathway. Breast Cancer Res..

[173] Tena-Sempere M., Manna P.R., Zhang F.P., Pinilla L., Gonzalez L.C., Dieguez C., Huhtaniemi I., Aguilar E. (2001). Molecular mechanisms of leptin action in adult rat testis: Potential targets for leptin-induced inhibition of steroidogenesis and pattern of leptin receptor messenger ribonucleic acid expression. J. Endocrinol..

[174] Tena-Sempere M., Barreiro M.L. (2002). Leptin in male reproduction: The testis paradigm. Mol. Cell. Endocrinol..

[175] Isidori A.M., Strollo F., More M., Caprio M., Aversa A., Moretti C., Frajese G., Riondino G., Fabbri A. (2000). Leptin and aging: Correlation with endocrine changes in male and female healthy adult populations of different body weights. J. Clin. Endocrinol. Metab..

[176] Panza S., Gelsomino L., Malivindi R., Rago V., Barone I., Giordano C., Giordano F., Leggio A., Comande A., Liguori A. (2019). Leptin Receptor as a Potential Target to Inhibit Human Testicular Seminoma Growth. Am. J. Pathol..

[177] Ni K., Steger K., Yang H., Wang H., Hu K., Chen B. (2015). Expression and role of leptin under hypoxic conditions in human testis: Organotypic in vitro culture experiment and clinical study on patients with varicocele. J. Urol..

[178] Ni F.D., Hao S.L., Yang W.X. (2020). Molecular insights into hormone regulation via signaling pathways in Sertoli cells: With discussion on infertility and testicular tumor. Gene.

[179] Gupta A., Herman Y., Ayers C., Beg M.S., Lakoski S.G., Abdullah S.M., Johnson D.H., Neeland I.J. (2016). Plasma Leptin Levels and Risk of Incident Cancer: Results from the Dallas Heart Study. PLoS ONE.

[180] Roelfsema F., Kok P., Veldhuis J.D., Pijl H. (2011). Altered multihormone synchrony in obese patients with polycystic ovary syndrome. Metabolism.

[181] Pehlivanov B., Mitkov M. (2009). Serum leptin levels correlate with clinical and biochemical indices of insulin resistance in women with polycystic ovary syndrome. Eur. J. Contracept. Reprod. Health Care.

[182] Panidis D., Rousso D., Kourtis A., Tsimas V., Papathanasiou K., Makedos G. (2003). Serum leptin levels in normal-weight and overweight women with polycystic ovary syndrome. Clin. Exp. Obstet. Gynecol..

[183] Oral E.A., Ruiz E., Andewelt A., Sebring N., Wagner A.J., Depaoli A.M., Gorden P. (2002). Effect of leptin replacement on pituitary hormone regulation in patients with severe lipodystrophy. J. Clin. Endocrinol. Metab..

[184] Iwasa T., Matsuzaki T., Tungalagsuvd A., Munkhzaya M., Kuwahara A., Yasui T., Irahara M. (2015). LH and testosterone production are more sensitive to the suppressive effects of food deprivation in prenatally undernourished male rats. Int. J. Dev. Neurosci..

[185] Ibrahim M.K., Alobaidi A.H.A. (2021). Evaluation of the Role of Ghrelin and Leptin as Biochemical Markers in Female with Polycystic Ovarian Syndrome. Anti-Inflamm. Anti-Allergy Agents Med. Chem..

[186] Gregoraszczuk E.L., Wojtowicz A.K., Ptak A., Nowak K. (2003). In vitro effect of leptin on steroids’ secretion by FSH- and LH-treated porcine small, medium and large preovulatory follicles. Reprod. Biol..

[187] Won E.T., Douros J.D., Hurt D.A., Borski R.J. (2016). Leptin stimulates hepatic growth hormone receptor and insulin-like growth factor gene expression in a teleost fish, the hybrid striped bass. Gen. Comp. Endocrinol..

[188] Krawczuk-Rybak M., Muszynska-Roslan K., Kitszel A., Sawicka-Zukowska M., Wolczynski S. (2005). Relationship between insulin-like growth factors (IGF-I and IGF-II), IGF-binding proteins (IGFBP-3, IGFBP-2), leptin and anthropometric parameters (height, body mass index) during antileukaemic treatment in children. Rocz Akad. Med. Bialymst..

[189] Hernandez M.I., Rossel K., Pena V., Cavada G., Avila A., Iniguez G., Mericq V. (2012). Leptin and IGF-I/II during the first weeks of life determine body composition at 2 years in infants born with very low birth weight. J. Pediatr. Endocrinol. Metab..

[190] Aiceles V., Gombar F., Da Fonte Ramos C. (2017). Hormonal and testicular changes in rats submitted to congenital hypothyroidism in early life. Mol. Cell. Endocrinol..

[191] Grasemann C., Wessels H.T., Knauer-Fischer S., Richter-Unruh A., Hauffa B.P. (2004). Increase of serum leptin after short-term pulsatile GnRH administration in children with delayed puberty. Eur. J. Endocrinol..

[192] Atamer A., Demir B., Bayhan G., Atamer Y., Ilhan N., Akkus Z. (2008). Serum levels of leptin and homocysteine in women with polycystic ovary syndrome and its relationship to endocrine, clinical and metabolic parameters. J. Int. Med. Res..

[193] Al Awadhi S.A., Al Khaldi R.M., Al Rammah T., Kapila K., Mojiminiyi O.A. (2012). Associations of adipokines & insulin resistance with sex steroids in patients with breast cancer. Indian J. Med. Res..

[194] Abel B.S., Muniyappa R., Stratton P., Skarulis M.C., Gorden P., Brown R.J. (2016). Effects of Recombinant Human Leptin (Metreleptin) on Nocturnal Luteinizing Hormone Secretion in Lipodystrophy Patients. Neuroendocrinology.

[195] Belobrajdic D.P., Frystyk J., Jeyaratnaganthan N., Espelund U., Flyvbjerg A., Clifton P.M., Noakes M. (2010). Moderate energy restriction-induced weight loss affects circulating IGF levels independent of dietary composition. Eur. J. Endocrinol..

[196] Zafeiridis A., Smilios I., Considine R.V., Tokmakidis S.P. (2003). Serum leptin responses after acute resistance exercise protocols. J. Appl. Physiol..

[197] Will K., Kuzinski J., Kalbe C., Palin M.F., Rehfeldt C. (2013). Effects of leptin and adiponectin on the growth of porcine myoblasts are associated with changes in p44/42 MAPK signaling. Domest. Anim. Endocrinol..

[198] Masuda S., Tanaka T., Masuzaki H., Nakao K., Taguchi S. (2014). Overexpression of leptin reduces the ratio of glycolytic to oxidative enzymatic activities without changing muscle fiber types in mouse skeletal muscle. Biol. Pharm. Bull..

[199] Higuchi T., Shirai N., Saito M., Suzuki H., Kagawa Y. (2008). Levels of plasma insulin, leptin and adiponectin, and activities of key enzymes in carbohydrate metabolism in skeletal muscle and liver in fasted ICR mice fed dietary n-3 polyunsaturated fatty acids. J. Nutr. Biochem..

[200] Ceddia R.B., William W.N., Curi R. (1999). Comparing effects of leptin and insulin on glucose metabolism in skeletal muscle: Evidence for an effect of leptin on glucose uptake and decarboxylation. Int. J. Obes. Relat. Metab. Disord..

[201] Ceddia R.B., William W.N., Curi R. (1998). Leptin increases glucose transport and utilization in skeletal muscle in vitro. Gen. Pharmacol..

[202] Berti L., Kellerer M., Capp E., Haring H.U. (1997). Leptin stimulates glucose transport and glycogen synthesis in C_2_C_12_ myotubes: Evidence for a P13-kinase mediated effect. Diabetologia.

[203] Sarmiento U., Benson B., Kaufman S., Ross L., Qi M., Scully S., DiPalma C. (1997). Morphologic and molecular changes induced by recombinant human leptin in the white and brown adipose tissues of C57BL/6 mice. Lab. Investig..

[204] Sainz N., Rodriguez A., Catalan V., Becerril S., Ramirez B., Lancha A., Burgos-Ramos E., Gomez-Ambrosi J., Fruhbeck G. (2012). Leptin reduces the expression and increases the phosphorylation of the negative regulators of GLUT4 traffic TBC1D1 and TBC1D4 in muscle of ob/ob mice. PLoS ONE.

[205] Sajan M.P., Ivey R.A., Lee M.C., Farese R.V. (2015). Hepatic insulin resistance in ob/ob mice involves increases in ceramide, aPKC activity, and selective impairment of Akt-dependent FoxO1 phosphorylation. J. Lipid Res..

[206] Balland E., Chen W., Dodd G.T., Conductier G., Coppari R., Tiganis T., Cowley M.A. (2019). Leptin Signaling in the Arcuate Nucleus Reduces Insulin’s Capacity to Suppress Hepatic Glucose Production in Obese Mice. Cell Rep..

[207] Lundasen T., Liao W., Angelin B., Rudling M. (2003). Leptin induces the hepatic high density lipoprotein receptor scavenger receptor B type I (SR-BI) but not cholesterol 7alpha-hydroxylase (Cyp7a1) in leptin-deficient (ob/ob) mice. J. Biol. Chem..

[208] Liang C.P., Tall A.R. (2001). Transcriptional profiling reveals global defects in energy metabolism, lipoprotein, and bile acid synthesis and transport with reversal by leptin treatment in ob/ob mouse liver. J. Biol. Chem..

[209] Kalaivanisailaja J., Manju V., Nalini N. (2003). Lipid profile in mice fed a high-fat diet after exogenous leptin administration. Pol. J. Pharmacol..

[210] Tariq S., Baig M., Tariq S., Shahzad M. (2017). Association of serum leptin with bone mineral density in postmenopausal osteoporotic females. Gynecol. Endocrinol..

[211] Roux C., Arabi A., Porcher R., Garnero P. (2003). Serum leptin as a determinant of bone resorption in healthy postmenopausal women. Bone.

[212] Petzel M. (2007). Action of leptin on bone and its relationship to menopause. Biomed. Pap. Med. Fac. Palacky Univ. Olomouc.

[213] Kocyigit H., Bal S., Atay A., Koseoglu M., Gurgan A. (2013). Plasma leptin values in postmenopausal women with osteoporosis. Bosn. J. Basic Med. Sci..

[214] Di Carlo C., Tommaselli G.A., Sammartino A., Bifulco G., Nasti A., Nappi C. (2004). Serum leptin levels and body composition in postmenopausal women: Effects of hormone therapy. Menopause.

[215] Bednarek-Tupikowska G., Filus A., Kuliczkowska-Plaksej J., Tupikowski K., Bohdanowicz-Pawlak A., Milewicz A. (2006). Serum leptin concentrations in pre- and postmenopausal women on sex hormone therapy. Gynecol. Endocrinol..

[216] Larcher F., Del Rio M., Serrano F., Segovia J.C., Ramirez A., Meana A., Page A., Abad J.L., Gonzalez M.A., Bueren J. (2001). A cutaneous gene therapy approach to human leptin deficiencies: Correction of the murine ob/ob phenotype using leptin-targeted keratinocyte grafts. FASEB J..

[217] Polyakova E.A., Mikhaylov E.N., Galagudza M.M., Shlyakhto E.V. (2021). Hyperleptinemia results in systemic inflammation and the exacerbation of ischemia-reperfusion myocardial injury. Heliyon.

[218] Leon-Cabrera S., Solis-Lozano L., Suarez-Alvarez K., Gonzalez-Chavez A., Bejar Y.L., Robles-Diaz G., Escobedo G. (2013). Hyperleptinemia is associated with parameters of low-grade systemic inflammation and metabolic dysfunction in obese human beings. Front. Integr. Neurosci..

[219] Van den Heuvel J.K., Eggels L., Van Rozen A.J., Luijendijk M.C., Fliers E., Kalsbeek A., Adan R.A., La Fleur S.E. (2014). Neuropeptide Y and leptin sensitivity is dependent on diet composition. J. Neuroendocrinol..

[220] Zhu L., Yang X., Li J., Jia X., Bai X., Zhao Y., Cheng W., Shu M., Zhu Y., Jin S. (2021). Leptin gene-targeted editing in ob/ob mouse adipose tissue based on the CRISPR/Cas9 system. J. Genet. Genom..

[221] Tsigos C., Kyrou I. (2002). Raptis SA: Monogenic forms of obesity and diabetes mellitus. J. Pediatr. Endocrinol. Metab..

[222] O’Rahilly S. (2007). Human obesity and insulin resistance: Lessons from experiments of nature. Biochem. Soc. Trans..

[223] Vega J.A., Salazar G., Hodgson M.I., Cataldo L.R., Valladares M., Obregon A.M., Santos J.L. (2016). Melanocortin-4 Receptor Gene Variation Is Associated with Eating Behavior in Chilean Adults. Ann. Nutr. Metab..

[224] Tao Y.X. (2009). Mutations in melanocortin-4 receptor and human obesity. Prog. Mol. Biol. Transl. Sci..

[225] Obradovic M., Sudar-Milovanovic E., Soskic S., Essack M., Arya S., Stewart A.J., Gojobori T., Isenovic E.R. (2021). Leptin and Obesity: Role and Clinical Implication. Front. Endocrinol.

[226] Hyogo H., Roy S., Paigen B., Cohen D.E. (2002). Leptin promotes biliary cholesterol elimination during weight loss in ob/ob mice by regulating the enterohepatic circulation of bile salts. J. Biol. Chem..

